# Written Exposure Therapy for PTSD Integrated with Cognitive Behavioral Coping Skills for Cannabis Use Disorder After Recent Sexual Assault: A Case Series

**DOI:** 10.3390/bs15070877

**Published:** 2025-06-27

**Authors:** Christine K. Hahn, Selime R. Salim, Emily L. Tilstra-Ferrell, Kathleen T. Brady, Brian P. Marx, Barbara O. Rothbaum, Michael E. Saladin, Constance Guille, Amanda K. Gilmore, Sudie E. Back

**Affiliations:** 1 Department of Psychiatry & Behavioral Sciences, Medical University of South Carolina, 67 President St. Suite 861, Charleston, SC 29425, USAferrelle@musc.edu (E.L.T.-F.);; 2 Department of Psychiatry, Boston University School of Medicine, 720 Harrison Avenue, Boston, MA 02118, USA; brian.marx@va.gov; 3 Emory School of Medicine, Emory University, 01 Dowman Dr., Atlanta, GA 30322, USA; 4 Department of Health Policy and Behavioral Sciences, Mark Chaffin Center for Healthy Development, School of Public Health, Georgia State University, Atlanta, GA 30303, USA; 5 Ralph H. Johnson Veterans Affairs Medical Center, 109 Bee St., Charleston, SC 29401, USA

**Keywords:** cannabis, posttraumatic stress disorder, sexual assault, integrated intervention

## Abstract

**Background/Objectives**: The co-occurrence of posttraumatic stress disorder (PTSD) and cannabis use disorder (CUD) symptoms is common following sexual assault, particularly among emerging adult women. CUD is associated with more severe PTSD symptoms and other mental health comorbidities including depression, suicidality, and emotion dysregulation. Addressing these issues concurrently soon after sexual assault could help decrease the risk for downstream negative health outcomes. Integrated trauma-focused interventions for PTSD and co-occurring substance use disorders have been shown to decrease PTSD severity and substance use. Yet, existing protocols are lengthy and have rarely been applied following recent trauma exposure or specifically to address CUD symptoms. **Methods**: This case series describes the application of Written Exposure Therapy (WET) for PTSD adapted to integrate cognitive-behavioral skills training for substance use among women following recent sexual assault. The adapted integrated intervention, Skills Training and Exposure for PTSD and Substance Misuse (STEPS), was delivered to three emerging adult women (age range = 19–25) who experienced recent sexual assault (weeks since assault range = 1–12 weeks). **Results**: This case series describes the novel intervention and examines clinical outcomes post-treatment and at the 1-month follow-up. Past-week PTSD symptoms (based on a clinical interview) and past-month cannabis use decreased among all participations after receiving STEPS. **Conclusions**: Preliminary findings from the case series provide new knowledge and insights regarding the application of STEPS following recent sexual assault among individuals with co-occurring PTSD and CUD. Therapeutic strategies for addressing PTSD and CUD concurrently and implications for future clinical research are discussed.

## 1. Introduction

Exposure to sexual assault (SA) is common and increases the risk for co-occurring posttraumatic stress disorder (PTSD) and substance use (Basile et al., 2022; Dworkin, 2020). Nearly one in two women in the United States (U.S.) experience unwanted sexual contact in their lifetime, and one in four experience completed or attempted rape. Most SA survivors report PTSD symptoms in the days following SA. Indeed, 74.5% meet the diagnostic PTSD criteria at one month post-SA, and 41.49% continue to meet the criteria 12 months post-SA (Dworkin et al., 2023). PTSD symptoms increase the likelihood that substance use and/or substance use disorders will begin or intensify, in part, because individuals may use substances to cope with SA-related PTSD (Bordieri et al., 2014; Haller & Chassin, 2014; Hicks et al., 2023). Cannabis is one of the most common substances used by individuals who experienced SA (Stewart et al., 2024). However, cannabis use may increase the risk of developing PTSD (Lee et al., 2018) and exacerbate PTSD symptoms over time (Metrik et al., 2020). Moreover, using substances to cope with PTSD symptoms after SA increases the risk for substance-related problems (Ullman et al., 2013). Research suggests that individuals who use cannabis to mitigate PTSD symptoms are more likely to experience greater cannabis-related problems (Farrelly et al., 2024) and develop cannabis use disorder (CUD; Kevorkian et al., 2015). Further, a recent systematic review of the clinical effects of cannabis on PTSD found that cannabis use was associated with risk for worsening PTSD symptoms among individuals with comorbid PTSD-CUD (Rodas et al., 2024). Taken together, these findings indicate that SA increases the risk for an adverse, cyclical relationship between PTSD symptoms and cannabis use, underscoring the importance of addressing these co-occurring issues following recent SA.

Cannabis is the most widely used non-prescribed drug in the U.S., with over 31 million people aged 12 and older reporting past-month cannabis use (Substance Abuse and Mental Health Administration, 2020). Regular cannabis use (i.e., daily or weekly) increases the risk for CUD. Indeed, 33% of people who regularly use cannabis are estimated to develop CUD (Leung et al., 2020). Women with CUD, relative to men with CUD, experience more interpersonal, legal, and health-related problems (i.e., hospitalization, emergency treatment, and sleep impairment; Gutkind et al., 2023). In addition, the frequency of cannabis use and the rates of CUD are higher among younger adults (i.e., ages 18–25) compared to older adults (Jeffers et al., 2021; Leung et al., 2020). Chronic cannabis use during this developmental period may put emerging adults at risk for poor cognitive functioning (Becker et al., 2018; Hall et al., 2020), potentially leading to negative consequences in educational and occupational functioning. These findings highlight the need for increased research on CUD treatment among at-risk emerging adult women.

SA is associated with increased risk for cannabis use in national samples (McCauley et al., 2010). Nearly a third (26.4–28%) of college women and 6.9–10.5% of household-residing women who have a history of SA use cannabis (Walsh et al., 2014). Comparably, only 8.9% of college and 2.7% of household-residing women who do not have a history of SA use cannabis. Emerging evidence also suggests that among people who use cannabis, those with SA histories use cannabis more frequently and report higher coping use motives than those with other types of trauma exposure (Stewart et al., 2024). In a daily diary study, experiencing sexual and/or physical violence from a dating partner predicted next-day cannabis use (but not alcohol use) among college women (Shorey et al., 2015), suggesting that cannabis may be used to cope with the immediate distress of violence. Indeed, among individuals who experienced recent SA, PTSD symptoms predicted cannabis use from six weeks to one year post-SA (Swain et al., 2020).

Excessive cannabis use is associated with numerous negative psychiatric outcomes, including other substance use disorders, mood disorders, suicide risk, lung illness, cardiovascular disease, and disability (Borges et al., 2016; Diep et al., 2022; Gutkind et al., 2021; Hasin et al., 2016; Hasin & Walsh, 2021), although more research is necessary to establish the causality of these associations. CUD is also linked to neurobiological changes that may increase the risk for developing PTSD after trauma exposure (e.g., impairment in executive functioning tasks associated with the prefrontal cortex; Cohen & Weinstein, 2018; Crean et al., 2011). Once exposed to trauma, symptoms of PTSD may also further exacerbate underlying neurobiological consequences of cannabis use, such as attention and emotional processing deficits (Kroon et al., 2021). The potential shared neurobiological risks underlying PTSD and CUD support that reducing co-occurring symptoms following recent SA has the potential to make a significant public health impact.

Effective, integrated psychosocial interventions applied soon after trauma exposure are needed to reduce PTSD and CUD symptoms and prevent the development of long-term, persistent mental health disorders. Integrated PTSD and substance use disorder treatments include trauma-focused interventions (i.e., focus on processing trauma-related memories, content, and emotions) and present-focused interventions (i.e., focus on promoting coping skills in present day situations). Meta-analyses of individual patient data from comorbid PTSD and substance use disorder treatment trials indicate that trauma-focused integrated interventions lead to greater improvements in substance use and PTSD symptoms than non-integrated interventions (Hien et al., 2023; Hill et al., 2024). Further, patients prefer integrated treatments compared to approaches that treat each disorder separately (Back et al., 2014a). However, integrated interventions are rarely applied shortly after trauma exposure and have seldom targeted cannabis use (e.g., Hill et al., 2024). Moreover, there is limited treatment research on comorbid PTSD and CUD, and integrated treatments are needed (Back et al., 2024). There are several provider-level barriers to implementing integrated treatments, including clinical considerations of delivering intervention components for both disorders (Mootz et al., 2022), clinician concerns that focusing on the trauma could exacerbate substance use (Gielen et al., 2014; Leeman et al., 2017), and limited provider training in CUD treatment (Bujarski et al., 2016). Dropout from integrated trauma-focused treatment for PTSD and substance use is also high, with estimates ranging from 30% to 70% (Kline et al., 2023). There are also barriers to care for substance use after SA, including stigma, shame, using substances to cope with trauma-related symptoms, and a lack of information or access to substance use treatments (Hahn et al., 2023). In line with these reported barriers, higher levels of substance use are related to lower mental health service use among those exposed to recent trauma (Van den Berk-Clark & Patterson Silver Wolf, 2017). Thus, there is an urgent need for an efficient, acceptable, accessible, and scalable model of care for addressing PTSD and CUD after SA.

Integrated interventions commonly use prolonged exposure (PE) to treat PTSD, in which patients repeatedly verbally recount their trauma memory during therapy sessions and systematically approach trauma reminders (i.e., places, activities, or situations) between sessions (Hien et al., 2023). Recent research has shown that Written Exposure Therapy (WET; Sloan & Marx, 2019) is an exposure modality that is noninferior to PE (Sloan et al., 2023). In WET (Sloan & Marx, 2019) patients write about the traumatic event during their session. WET may be an ideal modality to extend to integrated interventions for PTSD and substance use disorders because it is briefer (5 sessions) than other trauma-focused treatments (e.g., 12 sessions) and does not requires completing assignments outside of sessions. Further, findings from a recent systematic review suggest that WET is well-tolerated by patients, as evidenced by overall low treatment dropout rates and lower dropout than other trauma-focused treatments (DeJesus et al., 2024). As such, WET has been proposed as particularly well-suited for integrated treatment for PTSD and substance use disorders (Kline et al., 2023). Preliminary research from pilot trials supports the use of WET with people who have co-occurring PTSD and substance use disorders. Specifically, 31% of patients in a residential substance use disorder treatment program (Schacht et al., 2023) and 73% of pregnant women in a specialized obstetrics–addictions clinic (83% had a history of sexual assault; Nillni et al., 2023) with co-occurring PTSD and substance use disorders no longer met the criteria for PTSD after receiving WET. In a recent pilot study of predominately male veterans in a residential substance use disorder treatment setting, WET delivered in a group format was also associated with significant decreases in PTSD symptoms post-treatment (Van Doren et al., 2025). Clinical trials evaluating WET among veterans in substance use disorder treatment are currently underway (Meshberg-Cohen et al., 2024). An essential next step in this line of research is to apply an integrated WET treatment model for PTSD and CUD to individuals after recent exposure to SA.

### Present Study

This case series describes the application of Skills Training and Exposure for PTSD and Substance Misuse (STEPS), an efficient, early intervention that integrates WET for PTSD and cognitive behavioral skills training for substance use, among three emerging adult women who reported recent SA (i.e., past 12 weeks) and CUD. A review of the cases provides new knowledge about adapting lengthier integrated interventions into brief protocols and applying them in the weeks following trauma exposure for PTSD and CUD. The current study also aims to evaluate changes in PTSD symptoms and past-month cannabis use from baseline to 1-month follow-up among three women who received STEPS after recent SA. Given the preliminary nature of the study, observed changes in our outcomes can help generate hypotheses for future clinical trials regarding the direction, magnitude, and timing of changes in PTSD symptoms and cannabis use in integrated treatment following recent SA.

## 2. Materials and Methods

The Institutional Review Board at (BLINDED) University approved all study procedures. Women participated in an open pilot trial study examining an integrated intervention called STEPS (CITATION MASKED). Individuals were recruited after receipt of an SA medical forensic exam. During the exam, forensic nurses reviewed a research consent form with patients; interested patients provided their information to be contacted about potential research studies. Research staff contacted interested women via phone to inform them about the study and to complete brief screening to determine initial eligibility (age, time since SA, and use of substances). Individuals who met initial eligibility were scheduled for a baseline appointment. Informed consent was completed prior to any study procedures being conducted. Participants had the option to take as much time as needed to review the consent form and ask questions before making the decision to participate. After informed consent was obtained, a baseline appointment was scheduled and completed. At the baseline visit additional eligibility criteria were assessed. The eligibility criteria included (a) being assigned female sex at birth; (b) having experienced SA in the past 12 weeks; (c) having clinically significant PTSD symptoms (score of 31 or greater on the PTSD Checklist for DSM-5 [PCL-5]), (Blevins et al., 2015; Weathers et al., 2013); and (d) reporting CUD (reporting at least two CUD symptoms in the past-year, established via a clinical interview at baseline). The exclusion criteria included a lack of any memory of the SA, significant withdrawal symptoms, a change in psychotropic medications in the last two weeks, and current psychosis, mania, or eating disorders that required a higher level of care.

This case series describes the treatment of three participants who completed STEPS and identified cannabis as the primary substance they wanted to reduce during treatment. Cases in which alcohol was the primary target are reported separately (Hahn et al., 2025). Demographics are aggregated across the participants to increase anonymity. The three participants in the present case series (n = 2 for non-Hispanic White and n = 1 for non-Hispanic Black) were cisgender, heterosexual women, with ages ranging from 19 to 25. Education levels ranged from completing high school to completing a four-year college degree. For all participants, the most recent SA involved completed rape, with an average time of six weeks since the assault (range = 1–12 weeks). Case information is presented to illustrate the application of STEPS to CUD. In compliance with the Health Insurance Portability and Accountability Act (HIPAA), information is de-identified. Following informed consent and baseline, participants completed STEPS and were evaluated post-treatment and at the 1-month follow-up appointment. Due to the high risk for suicide after SA (Dworkin et al., 2022a), all participants completed a safety plan with the study therapist before starting STEPS, regardless of current suicidal ideation. The safety plan was updated as needed throughout the treatment. This study was pre-registered with clinicaltrials.gov, and relevant data are available through the National Institute on Alcohol Abuse and Alcoholism (NIAAA) data archive.

### 2.1. Measures

#### 2.1.1. PTSD

The past-week version of the PCL-5 was used to assess eligibility for inclusion at baseline. The PCL-5 was also administered prior to each STEPS session, at post-treatment, and at the 1-month follow-up. The PCL-5 consists of 20 items that assess self-reported PTSD symptoms within four symptom clusters: re-experiencing, avoidance, negative alterations in cognitions and mood, and alterations in arousal and reactivity. Items are rated on a scale from 0 (not at all) to 4 (extremely), with higher total scores indicating greater PTSD symptom severity. The past-month version of the PCL-5 has demonstrated excellent psychometric properties and utility in measuring self-reported severity of PTSD symptoms in various populations (Forkus et al., 2023); however, it should not be used for diagnostic purposes (Bovin & Marx, 2023). The past-week version of the PCL-5 was used instead of the past-month version because the time since SA at baseline was less than 4 weeks for some participants. A cut-off score of 31 or greater was used for inclusion because it is considered positive for clinically significant PTSD symptoms in the past-month version of the PCL-5. A change of ≥10 points is considered a clinically significant change according to the past-month version of the PCL-5 (National Center for PTSD, 2024).

The past-week version of the Clinician-Administered PTSD Scale for DSM-5 (CAPS-5; Weathers et al., 2013, 2018) was administered to participants by master’s- and doctoral-level evaluators to measure PTSD symptom severity related to the recent SA at baseline, post-treatment, and 1-month follow-up. The CAPS-5 is a gold-standard clinical interview for the assessment of PTSD and consists of 20 items, rated on a scale from 0 (absent) to 4 (extreme/incapacitating). Assessors were trained in the CAPS-5. Inter-rater reliability (based on the presence or absence of PTSD symptoms and CAPS-5 severity scores) was established by scoring audio-recorded interviews rated by an expert evaluator. Assessors were members of the research team but not study therapists. A total severity score was created by summing the severity score of each of the 20 symptoms, with higher scores reflecting more severe PTSD symptoms. In a male military sample, a severity score of <8 post-treatment has been identified as a clinically significant symptom change (Marx et al., 2022).

#### 2.1.2. Substance Use

Clinical interviews (Mini-International Neuropsychiatric Interview (Lecrubier et al., 1997); Diagnostic Interview for Anxiety, Mood, and OCD and Related Neuropsychiatric Disorders (Tolin et al., 2016)) were conducted to assess for substance use disorder diagnosis and symptom severity at baseline. The clinical interviews were also used to assess mood and psychotic mental health diagnoses.

The Timeline Follow Back (TLFB; Sobell & Sobell, 1992) was used to assess cannabis use in the past 30 days at baseline. At each therapy appointment and at post-treatment, participants were asked to report on cannabis use over the past week or since their last therapy session. Past-30-day cannabis use was also assessed at the 1-month follow-up. The TLFB is a calendar-assisted interview on daily substance use and has excellent test–retest reliability and concurrent validity (Carey, 1997). The TLFB has been validated for the assessment of cannabis use (Robinson et al., 2014).

### 2.2. Integrated Intervention

STEPS (see Table 1 for session content) integrates components of WET (Sloan & Marx, 2019) and cognitive behavioral coping skills based on the cognitive behavioral therapy manual for substance use disorder, which is a manual adapted from NIAAA’s Project Match (National Institute on Alcohol Abuse and Alcoholism, 2003). We selected cognitive behavioral coping skills because they have been successfully used in more lengthy evidence-based integrated protocols to reduce co-occurring PTSD and substance use disorder symptoms (Back et al., 2014b). Although the skills were originally designed for alcohol use disorder, they have been successfully applied to CUD and other substances (Hill et al., 2024). The selected coping skills are also relevant to SA and can be applied in day-to-day life. For example, learning how to identify and cope with triggers for cannabis use is related to coping with triggers for PTSD symptoms (e.g., a PTSD intrusion can lead to urges to use cannabis and breathing helps one cope with the intrusion and related cravings).

Each session maintains the content of WET and involves 30 min of writing about the traumatic event following specific prompts; STEPS adds 20 min of skills training based on content and exercises outlined in the cognitive behavioral therapy for substance use disorder manual (National Institute on Alcohol Abuse and Alcoholism, 2003). As written in the WET manual, psychoeducation is provided during the first session on PTSD and the rationale for exposure. STEPS integrates additional psychoeducation about common reactions to SA and the co-occurrence of PTSD and substance use. Skills training is provided during the first 20 min of each session. During the latter half of the session, patients complete 30 min of writing following prompts. Consistent with the WET manual, before and after writing, participants rate their subjective units of distress (SUDS) on a 0 (“No distress”) to 100 (“The most distressed I have ever felt”) scale. SUDS ratings provide information to the therapist about engagement in writing and habituation. STEPS builds on the SUDS ratings by additionally having participants rate their craving for cannabis prior to and immediately following the writing assignment (e.g., “Right now, how strong is your desire to use cannabis?”) on a scale of 0 (“No craving at all”) to 100 (“Most intense craving you have ever felt”). The second SUDS rating of cannabis craving is used to gauge if cravings changed before and after processing the SA. Participants are encouraged at the end of each weekly session to approach rather than avoid trauma cues during the next week. They are also asked to identify their top three coping skills in the session and apply them as needed when cravings arise in their day-to-day life. Consistent with WET, there are no formal homework assignments (e.g., worksheets or scheduled activities).

A therapist guide and a patient workbook were created by the first author to facilitate the delivery of STEPS. Therapists could add 1–2 additional sessions if participants did not engage with the exposure sufficiently in the first 2 sessions (e.g., did not write about the trauma in detail). Additional sessions only focused on completing the previously assigned writing prompts, and no coping skill content was covered. Two of the participants in the case series completed the therapy with the first author, who is a licensed clinical psychologist and a developer of STEPS. Consultation was provided to the first author by experts in WET and cognitive behavioral therapy for substance use disorder. A clinical psychology postdoctoral fellow provided STEPS to one of the participants in the case series after receiving training in STEPS from the developer. The fellow met weekly with the first author for supervision and completed adherence checklists during the session to verify that all STEPS components were delivered. Both authors had previous training in delivering cognitive behavioral therapy interventions and completed either in-person or online WET training (i.e., Sloan & Marx, 2021).

## 3. Results

**Figure 1:**
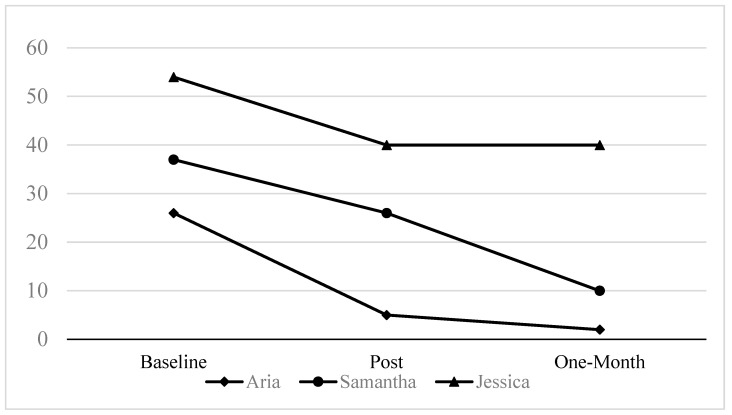
CAPS-5 PTSD symptoms.

**Figure 2:**
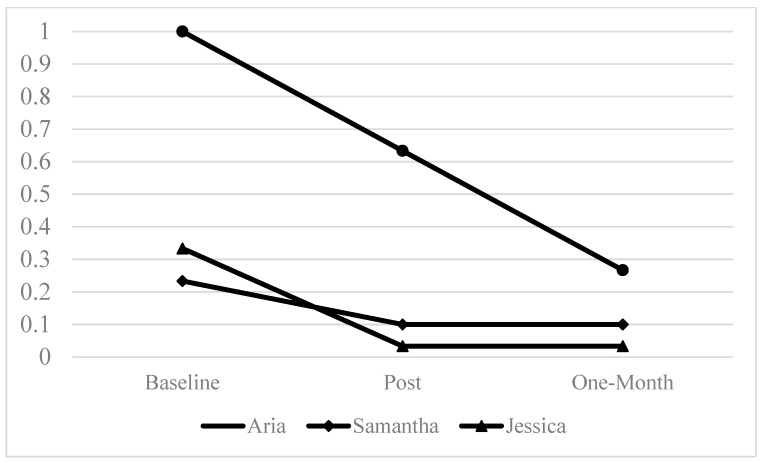
Percent of past 30 days cannabis was used.

All participants completed the entire STEPS protocol. There were no adverse events reported among any participants. Changes in our primary quantitative outcomes of past-30-day cannabis use and PTSD symptoms based on the CAPS-5 and PCL-5 are explained below. Figure 1 and Figure 2 display cannabis use and PTSD symptoms on the CAPS-5 at baseline, post-treatment, and 1-month follow-up.

### 3.1. Cannabis Outcomes

At baseline all participants met the criteria for CUD. The percent of days in which each participant used cannabis over the past 30 days ranged from 23% to 100%. At the follow-up, the percent of days each participant used cannabis over the past 30 days ranged from 1% to 27%. All participants reported a decrease in the percent of days in which they used cannabis over the past 30 days at the 1-month follow-up compared to baseline. Participants reported using cannabis 13% to 73% fewer days in the past 30 days at the 1-month follow-up compared to the 30 days prior to their baseline visit.

### 3.2. PTSD Symptom Outcomes

At baseline, two participants met the criteria for PTSD on the CAPS-5. The other participant endorsed elevated PTSD symptoms, but a PTSD diagnosis was not assigned because the SA occurred less than four weeks prior to the baseline visit. CAPS-5 scores decreased by 14 points or more from baseline to the follow-up among all participants. Two participants did not meet the criteria for PTSD at the follow-up; one participant met the PTSD criteria at the follow-up.

Per our inclusion criteria, at baseline all participants reported a PCL-5 score above 31 (cut-off for clinically significant symptoms; Blevins et al., 2015; Weathers et al., 2013). PCL-5 scores ranged widely across participants (range at baseline = 32 to 78). At the follow-up, two participants had minimal self-reported PTSD symptoms (PCL-5 scores < 10) and reported large decreases in symptom scores compared to baseline (decreases of 27 and 49 points, respectively). However, one participant continued to report high PTSD symptoms at the follow-up (decrease of three points compared to baseline).

### 3.3. Case Series

The course of treatment of the three participants who received STEPS as part of an open pilot trial is described in detail below. Participant outcomes are described with pseudonyms to maintain confidentiality. The first case includes additional details to illustrate how the components of the intervention are delivered.

#### 3.3.1. Case 1: Aria

**Background Information:** Aria experienced SA perpetrated by an acquaintance at her home after meeting him at a bar 12 weeks prior to the baseline visit. She sought a forensic exam and reported the rape to the police. At baseline, Aria described that prior to age 18, peers had attempted to engage in sexual acts with her through sexual coercion, and on one occasion a peer raped her. Aria stated that the most recent SA was currently the most stressful traumatic event she experienced. Aria reported daily cannabis use (~1.5 g per day). She described that the cannabis helped her cope with memories of the SA. Aria’s baseline self-reported PTSD symptoms (PCL-5 = 59) were elevated, and she met the criteria for PTSD on the CAPS-5 (severity score = 37). Aria reported some experiences of depersonalization since the SA on the CAPS-5. She denied suicidal ideation, plan, or intent.

**Session One:** At session one, the therapist and Aria discussed psychoeducation about common reactions to SA and the rationale for exposure-based writing. The therapist asked Aria how the SA she experienced and PTSD symptoms were related to her cannabis use. Aria described using cannabis before the SA in social contexts. Since the SA she had begun to use cannabis to help her fall asleep and decrease stress. The therapist used motivational interviewing to help Aria identify her treatment goals. Aria was asked to rate her readiness to reduce her cannabis use on a 10-point scale (1 = no at all ready; 10 = very ready). She reported strong motivation to reduce cannabis use (9/10 motivation). The therapist asked her why she rated her readiness to change as a nine instead of a lower number. Aria identified that reducing cannabis use would help her move toward her future career goals and improve her physical health. During the exposure-based writing, she was strongly engaged, which was indicated by tearfulness and self-report during the post-writing discussion. Her SUDS increased from pre-writing (SUDS = 60) to post-writing (SUDS = 100), which also indicated engagement. Her cannabis craving also increased from 50 pre-writing to 90 post-writing. The therapist instilled hope and validated that although engaging in exposure is difficult, it will help her benefit from treatment. The therapist used an exercise analogy (i.e., the first few weeks of exercise are difficult, but continued practice leads to increased strength and reduced soreness) to encourage future session attendance. Finally, the therapist praised her commitment to her health and encouraged her to allow trauma-related thoughts, feelings, and behaviors to be present without avoidance over the next week.

**Session Two:** At session two, Aria reported that she allowed herself to have some thoughts about the SA for a few minutes over the last week. She also tried to allow herself to experience emotions as they arose instead of using cannabis to cope. On a few occasions, she described allowing herself to experience difficult emotions without using cannabis. The therapist helped Aria identify triggers for cannabis use. She identified two triggers: trauma-related thoughts and feelings of anxiety. Skills for coping with triggers to use cannabis were reviewed including removing triggers, decision delay, positive alternative activities, and deep breathing. Aria also identified strategies to reduce cannabis use by altering her home environment (e.g., placing the cannabis in a difficult to access location and asking her roommate to keep her bong) and reported willingness to try decision delay (i.e., waiting 10 min before initiating any use upon craving). She identified three positive alternative activities to engage in when having cannabis cravings: (1) calling her sister, (2) going for a jog, and (3) going to a social outing with friends (e.g., bowling). The therapist led Aria in a brief, deep breathing exercise and encouraged her to use it when experiencing triggers related to SA and cannabis use. Aria reported finding this skill useful. The therapist provided feedback on her written exposure exercise from session one and reinforced her for including details and emotions (e.g., how the perpetrator looked during the rape, feelings of terror, and physical sensations). She was encouraged to continue writing and include in depth thoughts and feelings when writing in response to the prompt at session two. She conducted the exposure exercise during session two for 30 min. Upon completion of the writing exercise, Aria reported that the writing assignment was a “little bit easier” this session. Indeed, her SUDS only increased by seven points post-writing (50 pre-writing to 57 post-writing). She also did not report any cannabis craving before or after the writing. She reported feeling more intense anger during the writing session. Although no formal homework was assigned, Aria was encouraged to practice positive activities and deep breathing and experience SA-related thoughts and feelings without avoiding them.

**Session Three:** At session three, Aria reported allowing herself to have thoughts of the SA over the last week without avoiding them. She also reported reductions in past-week cannabis use. The therapist reviewed information about automatic thoughts and how certain thoughts often precede cannabis use. The therapist introduced several common unhelpful thinking patterns related to cannabis use (e.g., thinking cannabis will ease pain, make it easier to socialize, or make it easier to deal with a crisis). Next, Aria identified her own thought patterns. She recognized often having thoughts that cannabis will improve her mood. The therapist reviewed cognitive coping strategies for substance use including (1) identifying pros of not using cannabis and cons of cannabis use; (2) recognizing successes; and (3) developing alternative, helpful thoughts to replace unhelpful thoughts. Aria was provided with feedback during her session two written exposure exercise. She was praised for her willingness to write about emotional and sensory experiences during the SA and encouraged to continue writing such details in response to the next prompt. She wrote for 30 minutes following the prompt from session three. Her SUDS and cannabis cravings were similar to those reported in session two. Aria was encouraged to use coping skills over the next week outside of the session and to approach, rather than avoid, SA-related thoughts and feelings.

**Session Four:** At session four, Aria reported significant improvements including regularly allowing herself to have SA-related thoughts, feelings, and images without using cannabis to avoid the thoughts. Next, the therapist introduced the problem-solving skill and provided the rationale for the skill (i.e., having problems without skills for identifying solutions can increase frustration and, relatedly, cannabis use). The first step of problem-solving was reviewed (problem recognition). Aria identified cues from her body, thoughts, feelings, and behaviors (e.g., tension, racing thoughts, agitation, and withdrawal) that may signal the presence of a problem. Aria then was asked to identify a specific current problem in any life domain (e.g., interpersonal relationships and work). Aria selected to focus on the problem of using cannabis. The therapist helped her make the problem more concrete, and Aria reported using cannabis when she feels distressed at times, particularly in the evening. The therapist guided Aria through the next three steps of problem-solving: brainstorming various solutions, selecting the most promising approach, and taking action (i.e., if the strategy does not work, evaluate the plan and identify new solutions if needed). After skills training, the therapist provided feedback on the writing from session three and praised her for writing about the worst part of the SA. The therapist explained the session four writing prompt. Aria was encouraged to continue writing about the worst part of the SA in detail and to begin writing about the impact of the event on her beliefs about her life, the meaning of life, and relationships with others. As expected in this stage of treatment, her pre- and post-writing SUDS were low (0 and 15, respectively), and she continued to report no cannabis craving. After the writing exercise, Aria was encouraged to use problem-solving skills to reduce cannabis use and was instructed to not avoid any SA-related thoughts, feelings, and images that may arise over the course of the week.

**Session Five:** During the final session, Aria reflected on her progress during the treatment and expressed pride in her improvements. She shared her belief that engaging in trauma-focused treatment was uniquely helpful in reducing her cannabis use. The therapist helped Aria prepare for the future by identifying potential future high-risk situations for cannabis use and creating a prevention plan. In her writing during session four, Aria described feelings of hopeless and anger due to a negative interaction with the detective assigned to her SA (i.e., dismissal and victim blaming related to the rape by the detective), which prompted increased trauma-related thoughts (e.g., “the world is a terrible place”). Aria wrote for thirty minutes in response to the prompt during session five. During the session five written exposure exercise, Aria was encouraged to continue exploring beliefs about trust and the impact of the event on her future. In session five, she wrote about the difficulty of processing the emotional impact of the SA and fear that the impact could worsen in the future. She emphasized her desire to continue to grow and tend to the things she cares about the most. Her pre- and post-writing SUDS were low (0 and 20, respectively), and she reported no cannabis craving.

**Treatment Outcomes:** Aria reported significant reductions in cannabis use after completing STEPS. She engaged in cannabis use on 100% of the past 30 days at baseline and engaged in cannabis use on 63.3% of the past 30 days at post-treatment. At follow-up, her cannabis use further decreased; she reported engaging in cannabis use on only 26.7% of the past 30 days. Aria’s PTSD symptoms also decreased significantly at post-treatment, as measured by the PCL-5 (score = 11) and the CAPS-5 interview (score = 5). Treatment gains were maintained at follow-up (PCL-5 = 9; CAPS-5 = 8). Aria no longer met the PTSD criteria at post-treatment or the follow-up.

#### 3.3.2. Case 2: Samantha

**Background Information:** Samantha reported SA by a male peer after being at a bar with friends one week prior to the baseline visit. Samantha sought an SA forensic exam but did not report the rape to the police or the Title IX office. She described also being raped by a different peer during adolescence. Samantha described that currently the recent SA was causing more stress than the previous SA. Samantha reported using cannabis several times per week. At baseline Samantha reported PTSD symptoms on the PCL-5 (score = 32) close to the cut-off for clinical significance. She endorsed all criteria of PTSD on the CAPS-5 (severity score = 37) and denied experiences of dissociation. However, PTSD was not diagnosed due to the SA occurring less than one month prior to baseline. She also met the criteria for major depressive disorder (MDD) at baseline. Samantha reported passive suicidal ideation (“I don’t want to be here”) which occurred a few times per month, without plan or intent. Her safety plan included asking a friend for emotional support and accessing emergency services if needed.

**Session One:** Samantha reported using cannabis to “take the edge off” at the end of the day. Initially, she described some ambivalence about reducing cannabis use and did not see the behavior as currently problematic. However, she also reported believing that if she continued to use cannabis after college graduation, that would be problematic. During motivational interviewing, she reported high motivation (10/10) to reduce cannabis use. She reported rating a 10 because it bothered her that she noticed using cannabis to feel better since the SA and that she did not want to develop a cannabis use problem or exacerbate her depressive symptoms. She described that it would also make her feel emotionally strong to be able to recover from the SA without using cannabis. Samantha wrote for thirty minutes following the session one prompt. Samantha appeared to be engaged during the written exposure exercise and was tearful at times. She reported moderate SUDS (50) and low cannabis craving (20) at both pre- and post-writing. Elevated SUDS are expected pre- and post-writing during initial sessions and suggest that Samantha allowed herself to experience her emotions and memories.

**Session Two:** Samantha reported allowing herself to feel emotions and notice thoughts related to the SA over the past week. Samantha identified several triggers for cannabis use including being around friends who use cannabis, desire to have fun, and feelings of boredom. The therapist reviewed strategies to cope with cravings. Samantha decided on strategies including informing her friends that she was taking a break from cannabis use and focusing on alternative positive activities (i.e., going to the beach and eating out) when experiencing cravings. The therapist provided validation and positive feedback for her session one written exposure, including praising her for providing details of the SA and delving into the emotions (e.g., disgust, overwhelm, fear, and angry) she experienced during the SA. Samantha wrote for thirty minutes following the session two prompt. Samantha reported no distress (SUDS = 0) before pre-writing. She became tearful during the writing and reported increased distress post-writing (SUDS = 40), suggesting that she appropriately engaged in the exposure. She reported no cannabis craving pre- or post-writing.

**Session Three:** Samantha reported allowing herself to feel and think about the SA over the last week. The therapist reviewed psychoeducation about the connection between high-risk thoughts and cannabis use. Next, Samantha identified several cons of using cannabis (e.g., feeling tired, eating more food than desired, and parents finding out) and pros of reducing use (e.g., more energy and feeling proud). Samantha identified several successes, including performing well on school exams and reducing the number of days she used cannabis. The therapist praised Samantha for her session two writing, particularly for including difficult details of the SA (e.g., what the perpetrator said and thoughts and feelings during the SA). Samantha wrote for thirty minutes following the session three prompt. She was encouraged to continue focusing on SA details and to include information about her thoughts and feelings during the moment in which she realized the SA was going to happen. She continued to report moderate SUDS pre-writing (50) and a slight increase in SUDS post-writing (60). She reported low cannabis cravings pre- and post-writing (10).

**Session Four:** Samantha identified a school-related topic (i.e., deciding whether to stay on campus during the summer) for practicing the problem-solving skill. The therapist helped Samantha to identify various problem-solving strategies during brainstorming, including taking a different perspective on the problem. The problem-solving skill helped her identify that she was avoiding going to certain places on campus which reminded her of the perpetrator and the SA. She decided that staying near the college would be helpful because she could connect with friends and learn to enjoy her college experience after the SA. The therapist praised Samantha for writing in depth about the details of the SA in session three. She was encouraged to write about her thoughts related to trust during the session four prompt, as she previously wrote that the betrayal from the perpetrator was impacting her ability to trust others. Samantha wrote for thirty minutes following the session four prompt. As expected by session four, her pre- and post-writing SUDS were lower than previous sessions (10 and 30, respectively). She reported no cannabis craving.

**Session Five:** Session five focused on planning for the future and identifying high-risk situations for cannabis use, including seeing the perpetrator, school-related stressors, and worsening MDD. The most effective coping strategies employed throughout the treatment were identified for her to use in response to future stressors. The therapist recommended that Samantha engage in continued treatment for depression due to reports of persistent anhedonia and occasional, passive SI. Samantha agreed to accept referrals for treatment in the community. The therapist provided feedback and praised her for exploring the impact of the SA on her beliefs about others (e.g., men and institutions) due to feeling betrayed by both the perpetrator and the college. In the final writing session, Samantha wrote about learning that she has the strength and ability to face future challenges. Although she wrote about still struggling with trust, she described hope that she would be able to build new relationships in the future. She expressed minimal distress during and after writing. Her SUDS were 0 pre-writing and increased to 30 post-writing; she reported no cannabis craving pre- or post-writing.

**Treatment Outcomes:** Samantha engaged in cannabis use on 23.3% of the past 30 days at baseline, which reduced to on only 10% of the past 30 days at both post-treatment and follow-up. Samantha’s PTSD symptoms decreased on both self-report (PCL = 9) and interview (CAPS-5 = 26) measures at post-treatment. Treatment gains were maintained at the 1-month follow-up (PCL-5 = 5; CAPS-5 = 22). Samantha did not meet the criteria for PTSD at post-treatment or follow-up.

#### 3.3.3. Case 3: Jessica

**Background Information:** Jessica sought treatment after being involuntarily drugged and raped by a friend during a party six weeks prior to the baseline visit. She reported the SA to the police. Jessica reported experiencing SA perpetrated by an adult when she was a child. She described the most recent SA as the current most stressful life event she experienced. At baseline, she reported using cannabis on 33.3% of the past 30 days. She had high self-reported PTSD symptoms (PCL-5 = 73) and met the criteria for PTSD on the CAPS-5 (total score = 54). She reported struggling with daily depersonalization (e.g., episodes when she feels like she is not herself and time and her body seem to be moving slowly) and derealization (e.g., episodes when her surroundings feel unreal). Jessica met the criteria for MDD and reported suicidal ideation with a plan (e.g., driving into a tree) but did not intend to act on these thoughts. Jessica’s suicidal thoughts continued throughout the course of treatment. Thorough suicide risk assessments and safety planning were conducted at each visit prior to completing the STEPS therapy protocol.

**Session One:** Jessica reported using cannabis to have fun and relax with her partner and friends. She was ambivalent about reducing cannabis use (6/10 motivation) and reported that her primary motivation for reducing use was to pass drug tests for work. Through motivational interviewing, she elaborated on reasons for decreasing cannabis use such as facilitating emotional recovery from the SA. Jessica wrote for thirty minutes in response to the session one prompt. During the written exposure exercise, Jessica became highly distressed. The therapist provided grounding and encouragement to continue writing several times. Jessica was able to continue writing until the thirty-minute session was complete. She reported high SUDS pre- (80) and post-writing (100) as well as high cannabis craving pre- (70) and post-writing (100). After the writing, Jessica became withdrawn and made little eye contact with the therapist. The therapist normalized her distress level and attempted to instill hope by noting that her willingness to feel her emotions could help in her recovery.

**Session Two:** During session two, Jessica identified triggers for cannabis use. She identified triggers including intrusions related to the SA, physical pain related to a sports injury, and depressive symptoms (e.g., feeling down). In reviewing coping skills for triggers, she reported willingness to use decision delay when experiencing craving. She also identified several alternative positive activities (e.g., playing the guitar, video games, and exercise). However, Jessica described using exercise to cope with body dissatisfaction; she exercised if she believed she had consumed too many calories. The therapist briefly reviewed psychoeducation on the relationship between negative beliefs about one’s body and SA. The therapist encouraged Jessica to apply the cannabis use coping strategies to also refrain from overexercising. Jessica was praised for completing the writing in session one. In her first writing session, she focused on details related to the events leading up to the SA. She was given feedback to include more details about the moment the SA occurred. Jessica wrote for thirty minutes in response to the session two prompt. She continued to have difficulties engaging with the second written exposure exercise, stopping several times. The therapist used grounding and breathing techniques to help engage Jessica in the exercise. She continued to report high SUDS and craving pre- and post-writing. Jessica described that she distanced herself from her feelings to complete the exercise. She described feeling disconnected from herself and the experience.

**Additional Writing Exposure Session:** Jessica and the therapist discussed adding an additional session of writing in response to the session two prompt to further facilitate exposure and processing of the trauma. This decision was made because she had not provided full details about the SA, reported high levels of distress after writing, and reported avoiding emotions during the writing. Jessica agreed it would be helpful to repeat the session to ensure an adequate dose of exposure. The provider reviewed the rationale for emotionally engaging during the writing session and described the importance of including details about the SA. This session focused on the written exposure exercise only. Jessica reported high distress and craving pre and post-writing. (SUDs were 100 pre- and post-writing; craving was 90 pre- and 100 post-writing.) She also reported that she allowed herself to feel some of her emotions during this session.

**Session Three:** The therapist reviewed several examples of high-risk thinking for cannabis use. Jessica identified one high-risk thought she believed: that cannabis could be used like medicine to help with depressive symptoms. After reviewing coping skills (e.g., pros and cons and recognizing successes), she was able to identify more helpful thoughts about her ability to use healthier strategies to cope. She identified helpful strategies to cope with high-risk thoughts including spending time talking, playing games, and watching movies with her significant other. She identified successes related to cutting back on cannabis use over the past few weeks. She was praised for continuing therapy despite it being challenging. The therapist praised Jessica for writing about the SA in more detail in the additional session. Jessice was encouraged to continue writing about those details while allowing herself to experience emotions. Jessica continued to report SUDS of 100 pre- and post-writing as well as high craving pre- (90) and post-writing (100).

**Session Four:** Jessica identified an SA-related topic (i.e., contacting the detective in charge of her case to gather information about next steps) for practicing the problem-solving skill in session four. She described that this situation was a trigger for cannabis use. The therapist explained that the problem-solving skill may give Jessica a better sense of control over this stressor. However, given the systemic barriers to SA prosecution, problem-solving also focused on coping with not knowing if, and when, her case would be prosecuted. In her previous writing exercise, Jessica wrote about individuals (e.g., perpetrators) and systems (e.g., police) that evoked feelings of anger. She was encouraged to continue allowing herself to feel a range of emotions in addition to anger while writing about the worst part of the SA. She denied reductions in SUDS post-writing (pre- and post-writing SUDS = 100; pre-writing craving = 90 and post-writing craving = 100). She also reported difficulty allowing herself to feel trauma-related emotions.

**Session Five:** Session five focused on planning for high-risk situations in the future, including triggers related to her court case and being blamed by others for the SA. The therapist reviewed Jessica’s progress in decreasing cannabis use, elicited her strengths in accomplishing her substance use goals, and reviewed the benefits she had noticed from decreasing use. She was praised for the effort she put into written exposures. She wrote in response to the session five prompt for thirty minutes. Jessica’s final writing focused on her symptoms of depression, feeling trapped, and hopelessness (pre- and post-writing SUDS = 100; pre- and post-writing craving = 70). She described that her hope for the future was tied to the outcome of her SA case, such that she believed she could only recover if the perpetrator was held accountable. She expressed continued distress and impairment due to PTSD and depressive symptoms. The provider attempted to instill hope by describing other evidence-based treatments that were available. She was referred to dialectical behavioral therapy (DBT).

**Treatment Outcomes:** Jessica engaged in cannabis use 33.3% of the past 30 days at baseline and only engaged in cannabis use on 3.3% of the past 30 days at post-treatment and follow-up. Jessica’s PTSD symptoms remained elevated on the self-report measure of PTSD (PCL = 76); however, for the clinical interview, there was a decrease of 14 points in her PTSD symptom severity compared to baseline (CAPS-5 = 40). Jessica’s treatment response was similar at follow-up (PCL-5 = 70; CAPS-5 = 40), and Jessica continued to meet the criteria for PTSD.

## 4. Discussion

PTSD and CUD commonly co-occur after SA (Stewart et al., 2024) and are associated with risk for experiencing additional negative health outcomes including suicide (McFarlane et al., 2005) and revictimization (Walsh et al., 2014). There is an urgent need to establish efficient, accessible approaches to reduce and treat these common co-occurring symptoms following SA. The current case series describes STEPS, a novel five-session integrated intervention which applies WET and cognitive behavioral therapy skills to target PTSD symptoms and cannabis use. STEPS is the first integrated, exposure-based treatment designed to address co-occurring PTSD and cannabis use after recent SA. Potential advantages over existing treatment protocols include its brevity, tailoring for recent SA, and a lack of between-session homework. The current case series encourages further investigations of STEPS as a treatment option for PTSD and cannabis use following recent SA. In this case series, all three cases reported decreases in cannabis use post SA, which is typically a high-risk time for cannabis use to increase. Reductions in cannabis use were sustained one month post-treatment. Further, all participants had a clinically meaningful decrease in PTSD symptoms reported on the CAPS-5 (a decrease of 14 to 29 points across participants), and two out of three participants did not meet the criteria for PTSD at the one-month follow up. Although encouraging, these outcomes should be interpreted very cautiously as these results are based on a small sample of three participants. The primary aim of this case series was to describe the application of STEPS to co-occurring PTSD and cannabis use and examine individual treatment scores. Thus, conclusions about the efficacy of STEPS cannot be drawn.

Several important treatment considerations can be gleaned from this case series. MDD is a condition that is often comorbid with PTSD, CUD, and other substance use disorders (Bohn, 2003; Stefanidou et al., 2020; Ullman & Najdowski, 2009). Two participants in this case series reported MDD and suicidal ideation. Additional refinements to STEPS may be needed to tailor the protocol to individual patient needs regarding co-occurring depression. For instance, integrating behavioral activation and crisis management (Tyler et al., 2022) may be important to address co-occurring depression and suicide risk.

These cases illustrate common reasons for using cannabis among emerging adults who experienced SA including using cannabis to sleep, avoid traumatic stress symptoms, and socialize. There are theorized relationships between cannabis and recovery from trauma, including the potential for cannabinoids to facilitate memory consolidation and enhance fear extinction (Bedard-Gilligan et al., 2022). Recent studies found that delta-9-tetrahydrocannabinol (THC) administration before a Pavlovian fear conditioning paradigm may facilitate the activation of brain regions necessary for fear extinction learning in adults with PTSD (Zabik et al., 2023, 2024). However, it is not clear whether such findings apply to individuals with CUD given that other evidence suggests that chronic cannabis use may impair fear extinction (Papini et al., 2017). Concerns about the risks of using cannabinoids in the treatment of PTSD have also been raised (Maples-Keller et al., 2024). While several studies are testing cannabis as a treatment for PTSD, the available evidence does not support its use in treatment and suggests that it may increase the risk for worsening PTSD symptoms among those with comorbid PTSD and CUD (Rodas et al., 2024).

Most people who use cannabis are not interested in reducing their use or abstaining from it (Chauchard et al., 2013; Fernández-Artamendi et al., 2012); therefore, it is essential that treatment goals are made collaboratively. When initial treatment gains are made, patients may begin to see the benefit of decreased use and have greater self-efficacy to continue working towards continued reductions in use (Blevins et al., 2016). One way to reach individuals who report CUD but do not want to change their use may be to use integrated protocols that address co-occurring conditions (e.g., recovery from SA, PTSD, and MDD). Integrated protocols can increase individuals’ awareness into how their substance use may be associated with their other mental health concerns. For example, reflecting on the connection between cannabis use and SA-related distress assisted Samantha in formulating a goal of not using cannabis to cope with SA.

Two participants did not meet the criteria for PTSD at the follow-up. It is possible that they would have recovered naturally following the SA without intervention. However, most (71%) SA survivors meet the criteria for PTSD one month following the assault (Dworkin et al., 2023), a notably lower rate of natural recovery compared to survivors of other trauma types. Further, individuals with substance use disorders are less likely to naturally recover following trauma exposure because substance use promotes avoidance rather than the approach of trauma reminders (Hinojosa et al., 2024). Thus, given that both SA and substance use are predictors of less natural recovery after SA, intervening as early as possible post-SA may be an ideal approach to prevent the development of PTSD and co-occurring substance use disorders. Future research should explore the benefits and possible drawbacks of engaging in early interventions for PTSD in the initial weeks following SA.

One participant (Jessica) continued to meet the criteria for PTSD at the follow-up. She did not improve on self-reported PTSD symptoms on the PCL-5 but did have a decrease in symptom severity on the CAPS-5 (40 at follow-up compared to 54 at baseline). It is common for there to be discordance between PCL-5 and CAPS-5 symptom ratings, with PCL-5 items tending to be scored higher (Kramer et al., 2022). Although the CAPS-5 decrease suggested some improvement in symptom severity, Jessica’s symptoms were still elevated at the follow-up. Her writing focused heavily on feelings of anger towards friends, family, and formal systems that responded negatively to her disclosures of the SA. Such negative responses from informal and formal support systems are detrimental for survivors’ recovery (Dworkin et al., 2019) and likely exacerbated Jessica’s PTSD symptoms. Individuals faced with ongoing traumatic stressors (e.g., interaction with the criminal justice system and a pending legal case) may benefit from present-focused treatment tailored to their experiences and interventions for social support. For example, Dworkin and colleagues (Dworkin et al., 2022b) developed a supporter-focused early intervention to improve supporters’ ability to respond in more helpful ways to survivors after recent SA. This participant also reported depersonalization, derealization, and body dissatisfaction during STEPS. Transdiagnostic treatment approaches that can target numerous co-occurring problems related to underlying emotion regulation difficulties (e.g., substance use, disordered eating, suicidality, and dissociation) may be a useful approach for people with these co-occurring symptom presentations after SA. For example, a recent case series (Tilstra-Ferrell et al., 2024) applied WET in tandem with dialectical behavioral therapy skills for disordered eating with two SA survivors, one of whom experienced recent SA.

Emerging adulthood is a common time to initiate cannabis use (Pocuca et al., 2023) and a high-risk time for sexual violence (Morgan & Oudekerk, 2019; Dworkin et al., 2021). Untreated cannabis use early in life is associated with a long-term trajectory of use (Pocuca et al., 2023) and risk for a host of negative health outcomes throughout the lifespan (Karila et al., 2014). This underscores the importance of addressing co-occurring PTSD and CUD among emerging adults exposed to SA. STEPS was not specifically developed for emerging adults, but the results from this case series suggest that it may be an acceptable treatment for this population.

### Limitations and Future Directions

Future research is needed to rigorously assess the safety, acceptability, and feasibility of STEPS for PTSD and CUD following recent SA. It is important to keep in mind that natural recovery is common in the months following trauma (Steinert et al., 2015). Future research should examine rates of PTSD and CUD longitudinally following recent SA among people who receive STEPS in comparison to individuals who receive other interventions or no early interventions. We only followed participants for one month after STEPS, so additional research is needed to examine symptom change at longer follow-up periods. Although all participants met the criteria for CUD, there was variability in the frequency of reported cannabis use. It will be important for future work to determine how indicators of CUD severity (e.g., number of CUD symptoms experienced and length of CUD) may influence STEPS treatment outcomes.

Future research should also establish STEPS training procedures for clinicians. Previous studies of WET suggest that it requires less time for provider training compared to lengthier gold-standard PTSD treatments (Worley et al., 2020). Therefore, it may be more feasible to train providers in STEPS than other PTSD/CUD treatment options. The training protocol for STEPS in the research team’s ongoing research is for clinicians to attend a six-hour online training for WET (Sloan & Marx, 2021), a two-hour training in cognitive behavioral therapy skill content facilitated by the STEPS developer, and weekly supervision. Future research evaluating the most efficient training approaches for STEPS and other integrated interventions is needed to better understand clinician training needs and factors supporting treatment implementation.

The participants included in the current case series were all cisgender emerging adult women, with two identified as White and one identified as Black. Future research with larger and more diverse samples is needed to determine the appropriateness and effectiveness of STEPS for individuals with different identities and in other developmental stages. Future research on STEPS applied to address other substance use disorders besides CUD and evaluate its effectiveness in addressing polysubstance use is also needed. Research has noted different rates of cannabis use based on race/ethnicity, with American Indian/Alaska Native and Black/African American individuals having the highest rates of past-month use (Montgomery et al., 2022). Yet, systematic reviews indicate that racial/ethnic minorities remain under-represented in clinical trial research on substance use disorder treatment (Jordan et al., 2022), and culturally informed training and interventions are scarce (Hai et al., 2021). It is also important for clinicians to consider that there is disproportionate enforcement of cannabis use laws in Black/African American communities compared to White communities (Montgomery et al., 2022), which may understandably increase mistrust in systems of care and barriers to treatment access. Further, ongoing racial/ethnic discrimination and racial trauma are associated with both increased PTSD symptoms and cannabis use (Mattingly et al., 2023; Williams et al., 2021). Future revisions of the STEPS protocol may benefit from including additional components for empowerment and coping with ongoing experiences of racism and discrimination to improve treatment outcomes for racially minoritized patients (Holmes et al., 2024). Cultural adaptations to STEPS may be warranted to improve its application among minoritized individuals who experience systemic and institutional mistreatment and disproportionate legal consequences related to cannabis use (Sheehan et al., 2021). Future research on cultural adaptations should employ community-engaged implementation of science approaches (e.g., ADAPT-ITT (Escoffery et al., 2019); Model of Engaging Communities Collaboratively (McIlduff et al., 2020)) that amplify the voices and lived experiences of communities in the adaptation of interventions.

Future research is also needed to examine potential clinical predictors of response to STEPS such as trauma complexity, cumulative victimization, and dissociative symptoms. Importantly, the literature on transdiagnostic interventions that target numerous co-occurring problems due to underlying emotion regulation difficulties (e.g., substance use, disordered eating, suicidality, and dissociation) has recently grown (Dalgleish et al., 2020; Martin et al., 2018). Modular approaches like DBT-prolonged exposure (Harned et al., 2021), which involves teaching skills *prior* to implementing exposure for people who have multi-diagnostic presentations, difficulty engaging in treatment, and/or self-harm behavior, are also promising. Future research could examine the application and utility of transdiagnostic approaches for individuals who experience multiple co-occurring conditions after SA (e.g., co-occurring PTSD, CUD, eating disorder, and suicidality) in comparison to STEPS alone. STEPS could also be adapted to be delivered in a stage approach with DBT skills covered first for patients in need of stabilization prior to exposure.

## 5. Conclusions

In sum, STEPS is an integrated approach designed to address PTSD symptoms and CUD after recent SA. Future research formally evaluating STEPS should examine the efficacy of the integrated intervention for addressing PTSD and CUD simultaneously in larger samples following recent SA. Addressing PTSD and substance use post-SA has the potential to reduce the risk for sexual revictimization, long-term substance use, and a trajectory of chronic PTSD.

## Figures and Tables

**Table 1:** STEPS session content.

Session	CBT Skills Targeting CUD	WET Writing Prompt Targeting PTSD
1	Psychoeducation Motivational interviewing Goal setting	Write about details of sexual assault
2	Coping with urges and cravings to use cannabis	Write about details of sexual assault
3	Managing thoughts about cannabis use	Write about details of the worst part of sexual assault
4	Provider and patient select one of the following skills in collaboration based on need and relevance: Assertiveness, cannabis refusal skills, or problem-solving	Write about details of the worst part of sexual assault and the impact
5	Planning for high-risk situations involving cannabis	Write about the impact of sexual assault on one’s life and future

## Data Availability

The data presented in this study are available upon request from the corresponding author due to participants not providing consent for data to be submitted to national data archiving datasets.

## Abbreviations

The following abbreviations are used in this manuscript:

CBT	Cognitive Behavioral Therapy
CUD	Cannabis Use Disorder
PTSD	Posttraumatic Stress Disorder
SA	Sexual Assault
WET	Written Exposure Therapy
